# Prevalence of dysfunctional breathing associated with anxiety during the COVID-19 pandemic in Russia

**DOI:** 10.1192/j.eurpsy.2021.830

**Published:** 2021-08-13

**Authors:** J. Koniukhovskaia, O. Mitina, O. Stepanova, E. Dorokhov, E. Pervichko

**Affiliations:** Psychology, Lomonosov Moscow State University, Moscow, Russian Federation

## Abstract

**Introduction:**

The COVID-19 pandemic has become a situation of increased concern due to health threats and increased uncertainty. The risk of infection with the respiratory system coronavirus attracts increased attention to respiratory sensations. These two aspects can be beneficial grounds for the dysfunctional breathing-changes emergence in the breathing pattern that does not correspond to physiological needs.

**Objectives:**

To study the prevalence of dysfunctional breathing associated with anxiety during the COVID – 19 pandemic in Russia.

**Methods:**

The author’s socio-demographic questionnaire, the Naimigen Questionnaire, The State-Trait Anxiety Inventory were used. The survey was conducted online in May 2020. There were 582 participants (496 women&86 men) between the ages of 18 and 64.

**Results:**

The severity of dysfunctional breathing significantly correlated with the height of personal anxiety (r=0.488,p=0.000). Women are more likely than men to have dysfunctional breathing (18.1±9.6vs11.6 ±7.9;p=0.000) and have more expressed personal anxiety(26±10.5vs19.8 ±9.7; p=0.000). The age of respondents has an inverse correlation with personal anxiety (r=-0.147,p= 0.000), but not with dysfunctional breathing. Respondents who consider coronavirus to be a very dangerous trend to have dysfunctional breathing more than those who believe that the danger of coronavirus is exaggerated(18.1±10vs15.9 ±8.9;p=0.052).

**Conclusions:**

During the COVID-19 pandemic, the risk of dysfunctional breathing increases in a wide range of the population, especially among women. Since one of the dysfunctional breathing symptoms is a feeling of “difficulty inhaling”, anxious people may interpret this as shortness of breath in COVID-19, which may motivate them to seek medical help, thereby artificially increasing the burden on the health system during the COVID-19 pandemic.

**Conflict of interest:**

No significant relationships.

